# AYAs' online information and eHealth needs: A comparison with healthcare professionals' perceptions

**DOI:** 10.1002/cam4.5048

**Published:** 2022-07-25

**Authors:** Daniëlle L. van de Graaf, Carla Vlooswijk, Nadine Bol, Emiel J. Krahmer, Rhodé Bijlsma, Suzanne Kaal, Sophia H. E. Sleeman, Winette T. A. van der Graaf, Olga Husson, Mies C. van Eenbergen

**Affiliations:** ^1^ CoRPS ‐ Center of Research on Psychological disorders and Somatic diseases, Department of Medical and Clinical Psychology Tilburg University Tilburg The Netherlands; ^2^ Department of Research and Development Netherlands Comprehensive Cancer Organisation (IKNL) Utrecht The Netherlands; ^3^ Tilburg Center for Cognition and Communication (TiCC) Tilburg University Tilburg The Netherlands; ^4^ Department of Medical Oncology University Medical Center Utrecht The Netherlands; ^5^ Department of Medical Oncology Radboud University Medical Center Nijmegen The Netherlands; ^6^ Dutch AYA “Young and Cancer” Care Network Utrecht The Netherlands; ^7^ Department of Surgical Oncology Netherlands Cancer Institute Amsterdam The Netherlands; ^8^ Department of Medical Oncology Erasmus MC Cancer Institute, Erasmus MC Rotterdam The Netherlands; ^9^ Division of Psychosocial Research and Epidemiology Netherlands Cancer Institute Amsterdam The Netherlands; ^10^ Department of Surgical Oncology Erasmus MC Cancer Institute, Erasmus MC Rotterdam The Netherlands; ^11^ Division of Clinical Studies Institute of Cancer Research London UK

**Keywords:** adolescent, AYA, cancer, eHealth, healthcare professional, online information needs, young adult

## Abstract

**Background:**

Adolescents and young adults (AYAs) diagnosed with cancer fulfill their cancer‐related information needs often via the Internet. Healthcare professionals (HCPs) have a crucial role in guiding patients in finding appropriate online information and eHealth sources, a role that is often overlooked. Misperceptions of AYAs' needs by HCPs may lead to suboptimal guidance. We aimed to examine the extent to which AYAs' online information and eHealth needs corresponded with HCPs' perceptions of these needs.

**Methods:**

Two cross‐sectional online surveys (AYAs, *n* = 299; HCP, *n* = 80) on online information and eHealth needs were conducted. HCPs provided indications of their perceptions of AYA's needs.

**Results:**

AYAs reported significantly more online information needs compared with HCPs' perceptions regarding: survival rates (AYA = 69%, HCP = 35%, *p* < 0.001), treatment guidelines (AYA = 65%, HCP = 41%, *p* < 0.001), return of cancer (AYA = 76%, HCP = 59%, *p* = 0.004), “what can I do myself” (AYA = 68%, HCP = 54%, *p* = 0.029), and metastases (AYA = 64%, HCP = 50%, *p* = 0.040). Significantly more unmet eHealth needs were reported by AYAs compared with HCPs relating to access to own test results (AYA = 25, HCP = 0%, *p* < 0.001), request tests (AYA = 30%, HCP = 7%, *p* < 0.001), medical information (AYA = 22%, HCP = 0%, *p* = 0.001), e‐consult with nurses (AYA = 30%, HCP = 10%, *p* < 0.001), e‐consult with physicians (AYA = 38%, HCP = 13%, *p* = 0.001), and request prescriptions (AYA = 33%, HCP = 21%, *p* = 0.009).

**Conclusion:**

AYAs' online information and eHealth needs are partially discrepant with the impression HCPs have, which could result in insufficient guidance related to AYAs' needs. AYAs and HCPs should get guidance regarding where to find optimal information in a language they understand. This may contribute to AYAs' access, understanding, and satisfaction regarding online information and eHealth.

## Graphical abstract

This study examined the extent to which AYAs' online information and eHealth needs corresponded with HCPs CloseCurlyQuote; perceptions of these needs. Both needs of AYAs are shown to be partially discrepant with the impression of HCPs. AYAs and HCPs should get guidance regarding finding optimal information in a language they understand, which may contribute to AYAsCloseCurlyQuote; access, understanding, and satisfaction regarding online information and eHealth.

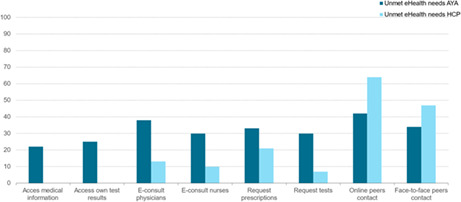

## 1 INTRODUCTION

Every year about 3800 adolescents and young adults (AYAs), aged 18–39 years, are newly diagnosed with any type of cancer in the Netherlands.^1^ The relative 5‐year survival rate for AYAs with cancer in the Netherlands is currently over 80% and is still rising. Even though AYAs are (much) younger than most cancer survivors, they are commonly treated in general oncology departments with little attention to their age‐specific questions.^2^, ^3^ However, research shows that AYAs have different needs related to their developmental phase of life such as individualized information, advice, communication, services, counseling (emotional and psychological), social support, and social relationships compared with older cancer survivors (e.g., study, work, fertility, family).^4^, ^5^, ^6^

AYAs are digital natives, which mean that they are native speakers of the digital language and pervasive users of technology.^7^ Next that, the amount of technology and Internet use, in general, has been rising (e.g., Skype, Instagram, WhatsApp).^8^ Therefore, their information needs often relate to the Internet, in addition to receiving information from healthcare professionals (HCP) directly.^9^, ^10^ However, previous research has shown that 50% of AYAs' online information needs are unmet, which negatively influences psychological distress, anxiety, and, in turn, quality of life,^11^, ^12^, ^13^, ^14^ but, importantly, it is unclear to what extent HCPs have an adequate perception of these needs. Information provision is of great importance in cancer care as fulfilled information needs may support adjustment to cancer, satisfaction, understanding, knowledge, patient participation, shared decision‐making, sense of disease‐control, acceptance, increasing compliance, rational expectations, self‐care, long‐term well‐being, and coping.^9^, ^15^, ^16^, ^17^, ^18^, ^19^

The way in which patients use the Internet has been described by Eysenbach^20^ and is, despite major changes in Internet usage, still perceived as a highly suitable model.^21^ The model includes the following four aspects of Internet usage: (1) content (i.e., online information), (2) communication, (3) communities, and (4) e‐commerce. However, since Eysenbach developed his model, e‐commerce (in a health setting) has been superseded by the broader concept of eHealth, which is the term Van Eenbergen et al. (2020) preferred, which facilitates self‐management.^21^ Self‐management refers to the use of electronic health records (including personal health information, medical history, medication, allergies, and hospital consult reports)^22^ or electronic diaries (e‐diary) in which patients are, for example, able to monitor pain intensity and medication adherence.^23^ However, in this study, we only address online information and eHealth since in this research, we only study self‐management without interactions with others.

With the increasing number of online information and eHealth possibilities, HCPs have the task to provide appropriate guidance and advice (e.g., web prescription, which includes guiding and advising on electronic media and online information) to AYAs because HCPs are the most reliable source of information for patients.^21^, ^24^, ^25^, ^26^, ^27^ A previous review has recognized that HCPs indeed have an important role in providing online information to AYAs.^28^ Other studies have shown that patients indeed indicate that they would appreciate web prescriptions given by their HCPs.^25^, ^26^, ^29^, ^30^, ^31^, ^32^, ^33^ However, HCPs are often uninformed about patients' online information needs. This is affected by HCPs' ability and willingness to talk about and provide online information as well as their understanding of AYAs’ needs.^28^ Thereby, online information seeking behavior may differ across disease stages,^34^, ^35^ of which HCPs may not be well aware either. This can lead to inappropriate or insufficient information provided to patients.^17^, ^24^, ^36^

Although various studies have examined AYAs' needs for online information and eHealth, little attention has been paid to a comparison of HCPs' perception of these needs. This is crucial as realistic perceptions of these needs contribute to appropriate guidance from HCPs. We, therefore, aim to examine (1) AYAs' online information seeking behavior and online information and eHealth needs; (2) HCPs' perceptions of AYAs' online information seeking behavior and online information and eHealth needs; and (3) the extent to which AYAs' indications and HCPs' perceptions correspond.

## 2 METHOD

### 2.1 Participants

**FIGURE 1:**
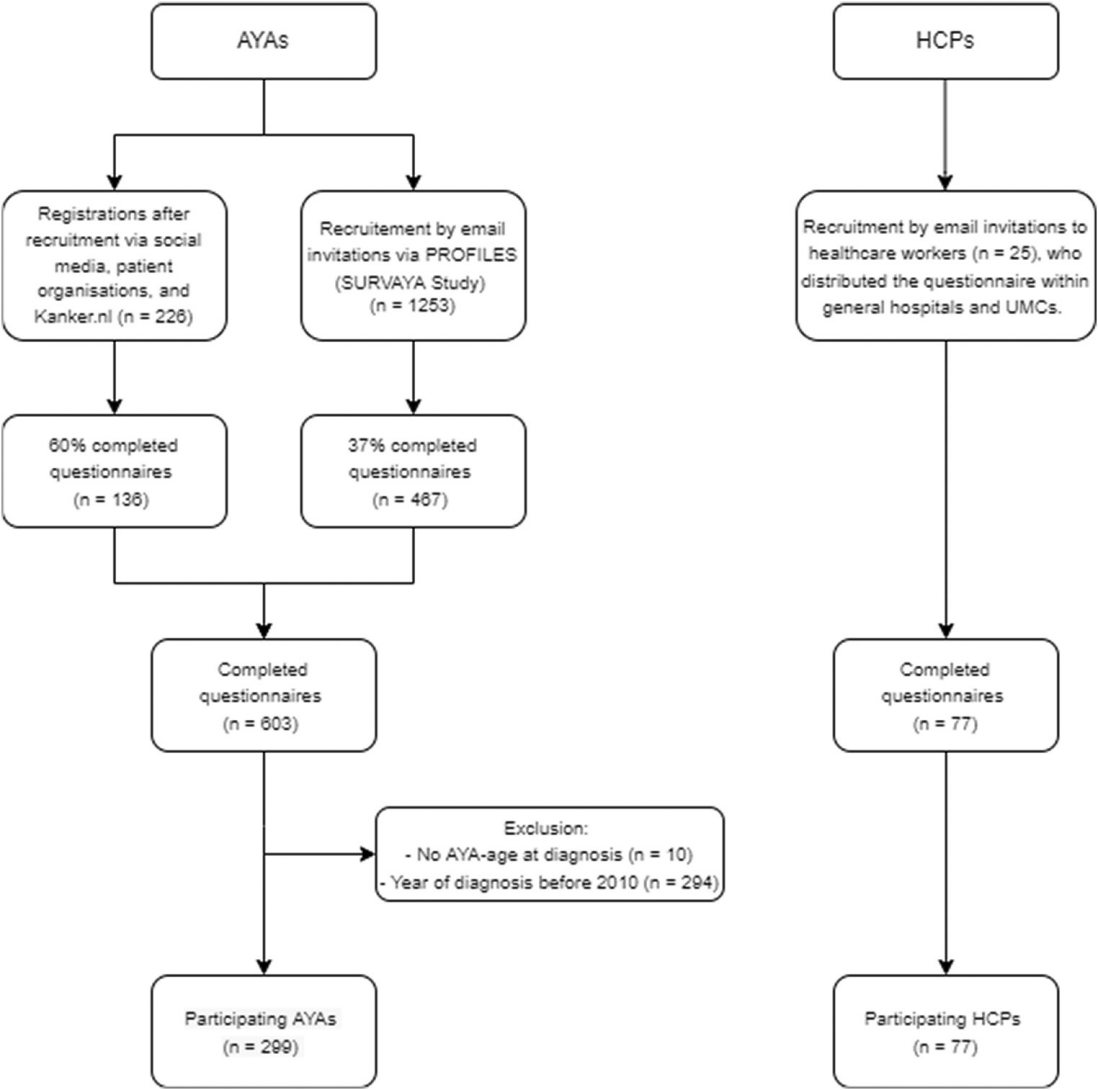
Flowchart of data collection.

Two cross‐sectional surveys on online information and eHealth were administered as follows: one among AYAs and one among HCPs. A flow chart of both data collections is presented in Figure 1. Data collection among AYAs was performed within PROFILES Registry (Patient Reported Outcomes Following Initial Treatment and Long Term Evaluation of Survivorship).^37^ AYAs were recruited through the distribution of the AYA questionnaire via Kanker.nl (the leading online platform in the Netherlands with information about cancer), newsletters of the Dutch Federation of Cancer Patient Organizations (NFK) and other patient organizations, social media, and three hospitals. Patients could participate if they were diagnosed with cancer during the AYA age. However, patients were excluded if cancer was diagnosed before 2010, as the Internet was less accessible and less common to use before 2010, and Internet use has been increasing since 2010.^38^

HCPs who were approached were HCPs from hospitals that the AYA care network in the Netherlands maintained contact with. Thus, these HCPs either had some form of AYA care or were willing to provide it. Participants received no compensation for participation. The questionnaire for HCPs was programmed in Qualtrics. HCPs were recruited via a mailing from the AYA healthcare network. The anonymity of patients and HCPs was guaranteed. This study was approved by the Research Ethics Review Committee of Tilburg School of Humanities and Digital Sciences (internal code: REDC 2019.104).

### 2.2 Measures

The AYA questionnaire was based on an existing questionnaire from the previous research.^21^ This questionnaire was adapted based on feedback from 6 AYAs (6 female, 26–33 years, diagnosed with cancer 0–6 years earlier) and AYA project leaders. A list of 67 items was generated, which was discussed with both AYAs and HCPs, after which adjustments were made. Adjustments included adding and removing questions, changing the order of questions, and adding minor nuances to questions.

Demographics including age, gender, education, year of diagnosis, type of cancer, and treatment modality were collected for AYAs. HCPs' demographics included gender, age at the time of questionnaire, profession, and employment or activity in the AYA expertise center or team.

We asked AYAs to indicate their online informationseeking behavior across disease phases (e.g., how often did you search for information about cancer on the Internet in the period that I had to wait for surgery/treatment?). HCPs had to indicate their perceptions of AYAs’ online information seeking behavior across disease phases (i.e., can you estimate how many of the AYAs search for information on the Internet?). Online information seeking behavior across the disease phases (1 = just before diagnosis, 2 = right after diagnosis, 3 = during treatment, and 4 = follow‐up) was measured with different values in HCPs (1 = all of them/many, 3 = none) compared with AYAs (1 = daily/several times a week, 3 = never). However, these questionnaires were compared in the analyses since HCPs are not able to estimate how often AYAs seek information. In this way, it was still possible to see to what extent the perceptions of HCPs correspond with the actual search behavior of patients.

Furthermore, needs regarding online information (e.g., did you search for information about fertility and wanting children after cancer during your illness and recovery period?) were assessed (0 = no, 1 = yes). HCPs were asked to make estimations about AYAs' online information needs (i.e., please mark which topics you think are important to AYAs, e.g., fertility and childbearing after cancer) (0 = no, 1 = yes).

Finally, eHealth needs (0 = no, 1 = yes) and possibilities (0 = no, 1 = sometimes, 2 = yes, and 3 = do not know) (e.g., what online options did you have and what wishes do you have in being able to request results of examinations?) were examined. HCPs were asked to indicate AYAs' eHealth needs (0 = no, 1 = yes) and possibilities (0 = no, 1 = yes, and 2 = do not know) (i.e., on each line, tick whether something is possible and whether you think AYAs would also find it desirable) (e.g., requesting test results).

Online information needs were considered as “unmet” when certain online information was indicated as needed but no possibilities were available. When it was indicated that no possibilities were available, this means that the person was not able to find certain online information. This does not automatically mean that the online information was not available.

### 2.3 Statistical analyses

Statistical analyses were conducted using SAS version 9.4 (SAS Institute, Cary, NC, 1999). The sociodemographic and clinical characteristics of the AYAS and HCPs were described as percentages or means and standard deviations. Searched topics on the Internet and eHealth needs were described as percentages. The searched topics on Internet were compared between AYAs and HCPs using chi‐square analyses (or Fisher's exact tests when sample sizes are small). Two‐sided *p* values of <0.05 were considered statistically significant. We decided not to conduct Bonferroni correction because of the exploratory nature of the study.

## 3 RESULTS

### 3.1 Sociodemographic and clinical characteristics

#### 3.1.1 AYAs

In total, 299 of 1479 invited AYAs (breast, 35%; lymphoma, 15%; gynecological cancer, 14%; testicular cancer, 7%; brain cancer, 5%; leukemia, 5%; gastrointestinal cancer, 4%; thyroid, 4%; sarcoma, 4%; skin cancer, 3%; and other, 4%) completed the questionnaire. The majority were female (78%), and the mean age at diagnosis was 31.8 years (SD = 5.7). The mean years since diagnosis was 6.1 (SD = 2.9). Most AYAs had a partner (78%) and finished college or university (64%). AYAs received multiple treatments, of which the most common treatments were surgery (72%), chemotherapy (71%), and radiotherapy (55%) (Table 1).

**TABLE 1:** Adolescents and young adults and healthcare professional characteristics.

Adolescents and young adults	*N*	%
Gender		
Female	234	78
Male	65	22
Age (at diagnosis), (mean [SD])	31.8 (5.7)	
18–25 years	45	15
25–39 years	254	85
Years since diagnose (mean ± SD)	6.1 (2.9)	
0–2 year(s)	51	17
>2–5 years	54	18
>5 years	194	65
Education level		
Primary school	0	0
Secondary school	108	36
College/university	190	64
Marital status (at time of questionnaire)		
Partner	233	78
Type of cancer^a^		
Brain cancer	16	5
Breast cancer	105	35
Gastrointestinal cancer	13	4
Gynacological cancer	43	14
Head and neck cancer	2	1
Leukemia	15	5
Lung cancer	3	1
Lymphoma	46	15
Sarcoma	9	3
Skin cancer	9	3
Testicular cancer	21	7
Thyroid cancer	12	4
Urological cancer	4	1
Other^b^	7	6
Treatment modality		
Surgery	215	72
Chemotherapy	211	71
Radiotherapy	164	55
Hormone therapy	56	19
Immunotherapy	33	11
Stem cell transplantation	15	5
Targeted therapy	8	3
Other^c^	7	2
**Health care professionals**		
Gender		
Female	65	84
Male	12	16
Age (at time of questionnaire), (mean [SD])	44.4 (10.1)	
Profession		
Medical specialist	25	32
Nurse	26	34
Nurse practitioner	16	21
Social worker	3	4
Sexologist	1	1
Psychologist	1	1
Other^d^	5	7
Employed in AYA expertise center	51	66
Active in AYA team	34	44

Abbreviation: AYA, Adolescents and young adults.

^a^ Total cancer types add up to more than 100%, as some AYAs reported having more than one type of tumor.

^b^ Neuroendocrine tumor, mesothelioma, trophoblast tumor, multiple myeloma, esthesioneuroblastoma, and thymus cancer.

^c^ Radioactive iodine therapy and no therapy or active surveillance.

^d^ Case managers, nurse consultants, and research physician.

#### 3.1.2 HCPs

In total, 77 HCPs (medical specialist, 32%; nurse, 34%; clinical nurse specialist, 21%; social worker, 4%; sexologist, 1%; psychologist, 1%; other, 7%) completed the questionnaire. The majority were female (84%) and their mean age were 44.4 years (SD = 10.1). The majority (66%) was employed in an AYA expertise center and 44% was active in an AYA multidisciplinary team (Table 1).

### 3.2 Searching for online information

#### 3.2.1 AYAs

**FIGURE 2:**
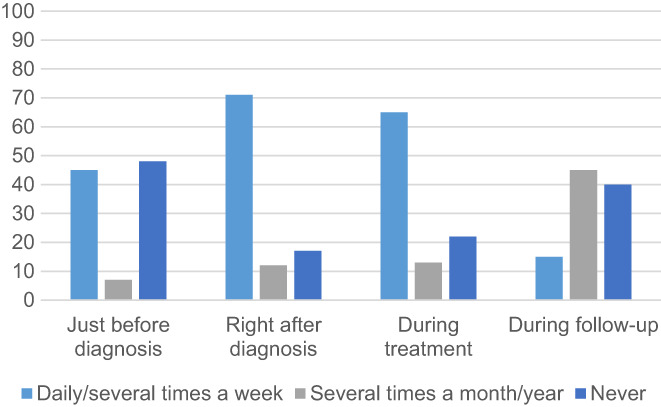
Search frequency for information about cancer on the Internet during different phases of disease according to adolescents and young adults.

Just before diagnosis, 45% searched the Internet for information about cancer daily to several times a week, whereas 48% never did. Directly after diagnosis, the number of patients who searched for information daily to several times a week increased to 71%, while those who never searched the Internet decreased to 17%. During treatment, 65% of patients searched the Internet daily to several times a week. During follow‐up, only 15% searched daily to several times a week and 40% did never search for information about cancer. Figure 2 shows when and how often AYAs searched the internet for information regarding cancer.

Furthermore, 52% of the AYAs were referred by HCPs to websites. Of those who were referred to websites, this regarded cancer information (34%), treatment consequences (13%), meeting AYAs online (12%), and/or relevant health apps (1%). Additionally, 17% of the AYAs asked for reliable sources of information. Of those who were referred to reliable sources of information, this regarded, cancer (14%), treatment consequences (6%), meeting AYAs online (2%), relevant health apps (1%), and/or other (10%) (e.g., patient association and nutrition).

#### 3.2.2 HCPs

**FIGURE 3:**
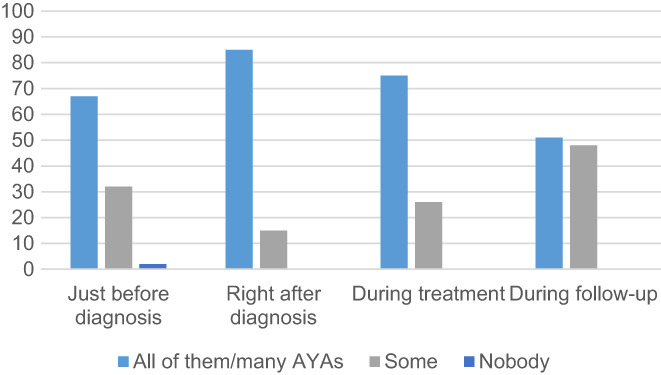
Search frequency for information about cancer on the Internet during different phases of disease according to healthcare professional perspectives.

Before diagnosis, HCPs reported that no (2%), some (32%), many (58%), or all (9%) AYAs searched on the Internet. In the period of waiting for treatment, HCPs reported that no (0%), some (26%), many (67%), and all (8%) AYAs searched on the Internet. After treatment (during the follow‐up), HCPs reported that no (0%), some (48%), many (45%), and all (6%) AYAs searched on the Internet. Figure 3 shows the perceptions of when and how often AYAs searched the Internet for information regarding cancer.

Additionally, 93% of the HCPs refer AYAs to the Internet to search for online information about cancer (sometimes, 41%; often, 37%; always, 16%). Next to that, HCPs reported that reliable sources of information were asked by no (4%), some (68%), many (28%), and all (0%) AYAs.

### 3.3 Searching for medical and psychosocial (age‐specific) topics on the Internet

#### 3.3.1 AYAs

The most frequently searched online medical information topics by AYAs related treatment consequences (79%), treatment (78%), late treatment effects (77%), return of cancer (76%), and survival rates (69%). Regarding psychosocial (age‐specific) information, the most frequently searched topics regarded lifestyle and nutrition (72%), activities and sport (70%), what can I do myself (68%), dealing with physical health problems (65%), and sexuality and intimacy (52%). Medical and psychosocial (age‐specific) topics that AYAs searched for on the Internet are shown in Table 2A and 2B, respectively.

**TABLE 2:** (A/B) Medical and psychosocial (age‐specific) topics searched for on Internet during and after treatment by AYAs and HCPS perspective.

	AYAs	HCP	*p*‐value
	%	%	
**(A) Medical topics**			
Consequences of treatment in general	79	87	0.15
Treatments	78	85	0.19
Late effects of treatment	77	88	**0.05**
Return of the same cancer	76	59	**0.01**
Survival rates	69	35	**<0.001**
Treatment guidelines	65	41	**<0.001**
Type of cancer	64	75	0.08
Metastases	64	50	**0.04**
Cancer genetics and heritability	60	82	**<0.001**
Chance of getting new cancer	51	53	0.82
Fertility and child wish	47	100	**<0.001**
Alternative or complementary therapies	32	56	**<0.001**
Finding a hospital	32	22	0.11
Trials and/or research	30	56	**<0.001**
Information about end of life	26	59	**<0.001**
Finding a doctor	25	25	1.00
Involvement treatment decision	21	84	**<0.001**
Doctor patient relationship	13	46	**<0.001**
Information about palliative treatment and/or palliative care	6	51	**<0.001**
**(B) Psychosocial (age‐specific) topics**			
Lifestyle and nutrition	72	62	0.12
Activities and sports	70	62	0.20
What can I do myself	68	54	**0.03**
How to deal with physical health problems (e.g., fatigue and pain)	65	63	0.83
Sexuality and intimacy	52	89	**<0.001**
How to deal with mental health problems (e.g., anxiety and depressive feelings)	50	71	**0.002**
Return to work and/or study	47	89	**<0.001**
Insurance and/or mortgage	44	95	**<0.001**
Improve satisfaction with your own body image after treatment	31	56	**<0.001**
Learn to stand up for yourself, regain self‐confidence	28	46	**0.01**
Learn to look at life in a positive way	26	30	0.46
Consequences for young family	25	98	**<0.001**
Deal with the feeling of lagging behind “healthy” peers	24	60	**<0.001**
Meeting possibilities peers	24	81	**<0.001**
Help for family and friends	20	56	**<0.001**
Spirituality	19	29	0.09
Deal with parents and/or family members	18	60	**<0.001**
Friendships	14	44	**<0.001**
Financial problems	13	76	**<0.001**
Establish relationships	9	63	**<0.001**
Religion	8	13	0.20

*Note*: Significant *p* values are in boldface.

#### 3.3.2 HCPs

According to HCPs, AYAs searched the Internet mostly relating fertility and child wish (100%), late effects of treatment (88%), consequences of treatment in general (87%), treatments (85%), and involvement in treatment decision (84%). Regarding psychosocial (age‐specific) information the most frequently searched topics by AYAs according to HCPs were consequences for a young family (98%), insurance and/or mortgage (95%), sexuality and intimacy (89%), return to work and/or study (89%) and meeting possibilities fellow sufferers (81%). HCP indications of their perceptions of AYAs' online information seeking regarding behavior regarding medical and psychosocial (age‐specific) topics are shown in Table 2 and 2B, respectively.

#### 3.3.3 Comparison of AYAs and HCPs

Compared with HCPs' perceptions, AYAs reported significantly more online information seeking regarding the topics return of cancer (AYA = 76%, HCP = 59%; *p* = 0.01), survival rates (AYA = 69%, HCP = 35%; *p* < 0.001), treatment guidelines (AYA = 65%, HCP = 41%; *p* < 0.001), metastases (AYA = 64%, HCP = 50%; *p* = 0.04), and what can I do myself (AYA = 68%, HCP = 54%; *p* = 0.03).

### 3.4 (Future) whishes regarding eHealth

#### 3.4.1 Comparison of AYAs and HCPs

**FIGURE 4:**
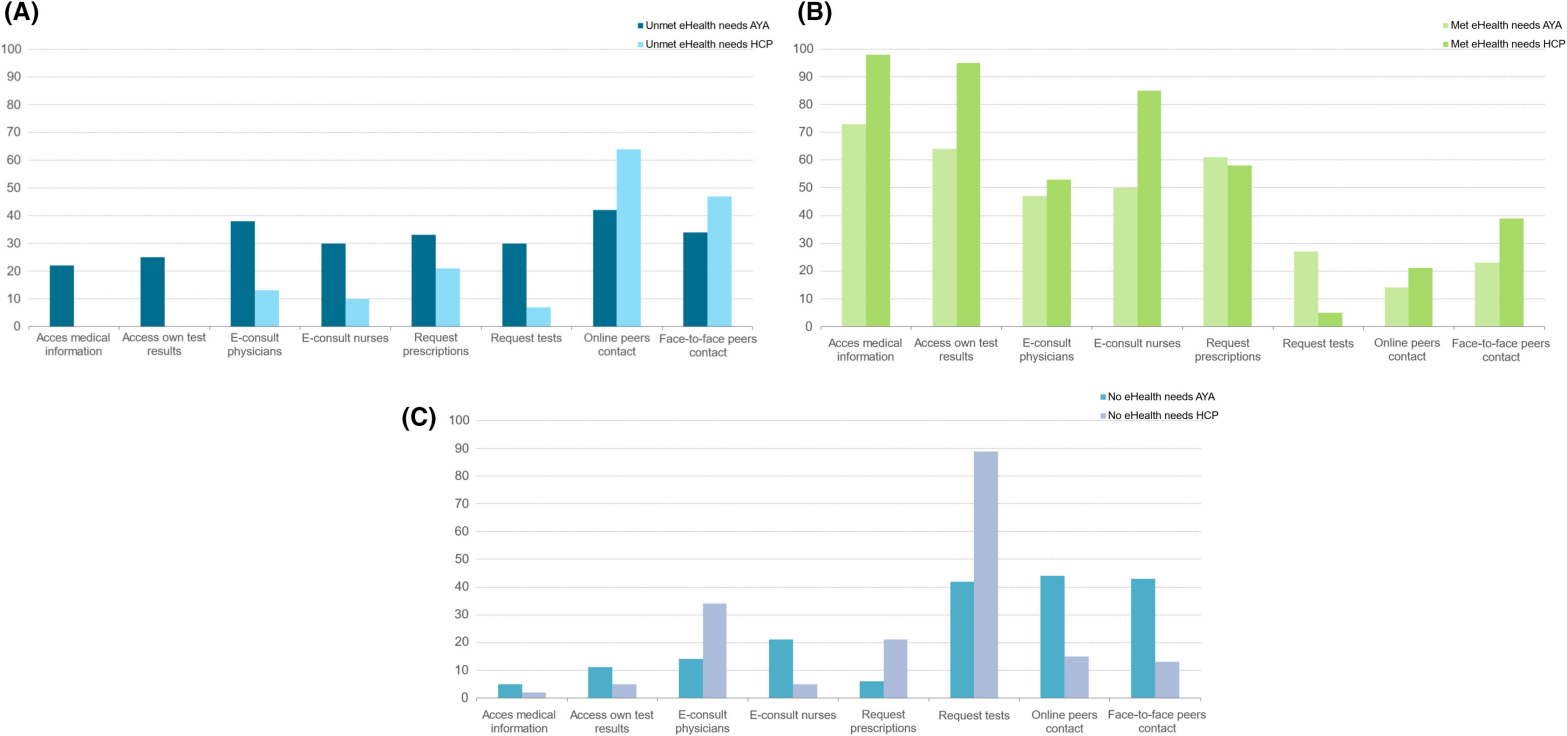
(A) Unmet eHealth needs by adolescents and young adult (AYA) and healthcare professional (HCP) perspectives. (B) Met eHealth needs from AYA and HCP perspectives. (C) No eHealth needs by AYA and HCP perspectives.

Furthermore, AYAs' eHealth wishes and needs were examined, as well as the relating perceptions of HCPs, of which the significant differences are shown in Figure 4A. AYAs reported significantly more unmet eHealth needs regarding access medical information (AYA = 22%, HCP = 0%; *p* < 0.001), access own test result (AYA = 25%, HCP = 0%; *p* < 0.001), e‐consult physicians (AYA = 38%, HCP = 13%; *p* = 0.001), e‐consult nurses (AYA = 30%, HCP = 10%; *p* < 0.001), request prescriptions (AYA = 33%, HCP = 21%; *p* = 0.01), and request tests (AYA = 30%, HCP = 7%; *p* < 0.001) compared with HCPs' perspectives of these needs. In contrast, it was found that HCPs overestimate AYAs' unmet eHealth needs regarding online peer contact (AYA = 47%, HCP = 64%; *p* = 0.002) and face‐to‐face peer contact (AYA = 34%, HCP = 42%; *p* = 0.004).

## 4 DISCUSSION

This study examined AYAs' online information and eHealth needs, HCPs' perceptions of these needs, and the extent to which AYAs' indications and HCPs' perceptions correspond. Results showed that AYAs mostly sought information directly after diagnosis and during the treatment phase, whereas HCPs expected AYAs to seek information in similar frequencies across the different phases. Furthermore, our results showed that HCPs underestimate online information seeking of AYAs regarding several medical (e.g., return of cancer, treatment guidelines, metastases) and psychological (i.e., what can I do myself) topics. HCPs also underestimated several eHealth needs of AYAs, such as assessing test results, e‐consults with physicians or nurses, and requesting tests or prescriptions. However, it was shown that HCP's overestimate eHealth needs regarding peer contact. Such underestimations and overestimations could result in a mismatch in online information and eHealth guidance and needs between HCPs and AYAs. As a result, HCPs may provide (1) too little information about certain topics because they think that AYAs read about them online and (2) too few suggestions where this kind of information can be found online. This is undesirable since meeting information needs is crucial for several health outcomes of patients with cancer (e.g., knowledge, understanding, shared decision‐making, long‐term well‐being, coping).^9^, ^15^, ^16^, ^17^, ^18^

It has been shown that there are inconsistencies between AYAs' needs for online information and eHealth and how HCPs perceive these needs, which may explain why the provision of information from HCPs to patients often does not match the needs of the patient.^24^, ^39^, ^40^ This could be explained by HCPs providing information that they think is important, instead of checking what the AYA finds important.^41^ In addition, this problem may be greater in this specific patient group due to the age difference between AYAs and HCPs (range 24–62 years), which appears to complicate communication between them.^42^, ^43^ Furthermore, previous research has shown that many HCPs experience difficulties in communicating and connecting with AYAs,^44^ which could indicate that during the consultations, the needed topics are therefore not discussed. It is important that HCPs are attentive to the patient's preferences and that they realize the urgency to guide AYAs so that AYAs are satisfied with the usage of appropriate online information and eHealth possibilities.^45^ To achieve this, AYAs and HCPs should get guidance regarding where to find optimal information in a language they understand. Guidance is essential in optimal online information use, to ensure that AYAs find reliable and complete information. One of the treating HCPs should refer to one place on the Internet, where all age‐tailored services and information are presented. In the Netherlands, this is currently being developed as part of the COMPRAYA study.^46^ Next to that, HCPs could refer patients more frequently to electronic health records. When necessary, HCPs should provide guidance in finding and using these sources to encourage optimal usage.

In addition, the type of online information needs to be indicated by AYAs and HCPs differs. AYAs seem to be mainly concerned about the cancer diagnosis and treatments they are currently in, including topics such as treatment, survival rates, lifestyle and nutrition, and activities and sports during treatment. This means that AYAs are primarily focused on survival and (re)starting life. Meanwhile, HCPs are also concerned about the future of AYAs (i.e. after treatment), which may include topics such as children, sexuality, finances, and resume work. It is important to emphasize that HCPs should actively contribute to highlighting this long‐term topics.^47^

Interestingly, AYAs report a high level of need for online and offline peer contact. AYAs report relatively high peer contact needs (almost 60%) compared with the average cancer population (around 10%–20%).^21^, ^48^ Therefore, it would also be advisable to allow for the possibility to meet peers online for information. This recognized relevance of peer support seems to have increased significantly in recent years from an HCPs' perspective, as it was previously seen as less relevant.^49^ To our knowledge, there is no research that has studied this. However, over the past 10 years, patient organizations in the Netherlands have been visibly active with topics such as cocreation, patient journeys, and shared decision‐making. The healthcare sector, and thus HCPs, is giving more substance to these topics in the last 10 years The growth in recognized relevance could be explained by the increasing relevance of patient advocacy. The opportunities for peer support that the Internet, and specifically social media, offer can also play a role in this. The fact that care providers are now well aware of the need for and importance of contact with peers may improve guidance for opportunities in peer contact.

### 4.1 Limitations

Several limitations of this study need to be acknowledged. First, since nearly 80% of the patient sample is female, this is not an adequate representation of the AYA patient group. This could possibly be explained by the high proportion of breast cancer patients. However, this may have led to partly nongeneralizable results for the general AYA population, since women report more information needs and seeking behavior compared with men.^36^, ^50^ Gender also influences the type of online information needs, as, for example, women have more interpersonal and emotional information needs, whereas men have more information needs regarding sexual function and fertility.^51^ The results must therefore be interpreted with caution. Second, this study mostly includes respondents with higher levels of education. Higher educated patients tend to have more information needs compared with lower educated patients.^52^, ^53^, ^54^, ^55^ Highly educated people are more self‐reliant and are therefore more likely to gather online information to help them take care of themselves, while patients with lower levels of education have higher support service needs, which means that they are more likely to use specific AYA care.^54^ Third, this study includes a native respondent group. This group may be more likely to search online for cancer information than ethnic minorities since people from ethnic minorities generally experience more difficulties and less trust in sources during online information seeking.^56^ Specific attention should be paid to this group because they are less proficient in the language. Therefore, careful and culturally appropriate consideration should be given to guidance regarding online information.^56^ Fourth, no comparison was made between the outcomes of HCPs who do and do not work in an AYA expertise center or AYA team. However, an increasing amount of AYA expertise centers emerge in the Netherlands, making it increasingly relevant.

## 5 CONCLUSION

It can be concluded that HCPs both overestimate and underestimate online information and eHealth needs of AYAs. This indicates that information that is provided does not always meet the patient's needs, while it is crucial to meet their needs since adequate information is vital for coping with cancer. It is advisable that AYAs and HCPs should get guidance regarding where to find optimal information in a language they understand. This may contribute to AYAs' access to, understanding of, and satisfaction with online information and eHealth.

## AUTHORS’ CONTRIBUTION

M.C. van Eenbergen, O. Husson, N. Bol, and S. Sleeman contributed to the study conception, design, and material preparation. Introduction, Method, and Discussion were written by D.L. van de Graaf. Analyses and Results were performed by C. Vlooswijk. Results were discussed by D.L. van de Graaf, M.C. van Eenbergen, C. Vlooswijk, and O. Husson. All authors commented on previous versions of the manuscript. The final version was read and approved by all authors.

## FUNDING INFORMATION

The COMPRAYA study was funded by an Infrastructural grant (#11788) from The Dutch Cancer Society (Amsterdam, The Netherlands). Dr. Olga Husson is supported by a personal research grant (VIDI; #198.007—Facing the unthinkable in the prime of life: Prevalence, risk factors, and mechanisms of impaired medical and psychosocial health outcomes among adolescents and young adults with cancer) of the Netherlands Organization for Scientific Research.

## CONFLICT OF INTEREST

None.

## Supporting information


Appendix S1

Appendix S2

## Data Availability

Data are available upon request pending approval by the authors.
