# Supplementary material for: AYAs' online information and eHealth needs: A comparison with healthcare professionals' perceptions
Source: Cancer Med. 2022 Jul 25;12(2):2016–26. doi: 10.1002/cam4.5048 (PMC9883566; doi:10.1002/cam4.5048)
Supplement: Supplementary file 1 — Appendix S1 [file CAM4-12-2016-s001.docx]

**Appendix A**

1. Have you been diagnosed with cancer before your 40th birthday?

- Yes (this group can proceed to the questionnaire)
- No (this group is excluded and cannot proceed with the questionnaire) 🡪 "Thank you very much for your interest in our study. This questionnaire is only intended for people who have been diagnosed with cancer before the age of 40, and because of this you are unfortunately not eligible to participate in this study."

1. What is your gender?

- Male
- Female
- Other

1. In what year were you first diagnosed with cancer? _______
2. What type(s) of cancer have you been treated for? (more answers possible)

- Cervical cancer
- Breast cancer
- Bone cancer
- Colorectal cancer
- Ovarian cancer
- Endometrial cancer
- Brain tumor
- Leukemia
- Lymphoma
- Melanoma
- Sarcoma
- Thyroid cancer
- Testicular cancer
- Other, namely _______

1. What treatments have you had? (more answers possible)

- Radiation
- Chemotherapy
- Targeted therapy
- Hormone therapy
- Immunotherapy
- Surgery
- Stem cell transplant
- Other, namely _______

1. What is your current marital status?

- I am married
- I am not married, but live with my partner
- I have a steady partner, but we live apart
- I have no partner
- Other, namely _______

1. What is the highest level of education you have completed?

- Primary School
- Primary vocational education (LBO)
- Secondary education (such as MAVO, MULO)
- Secondary (vocational) education (MBO)
- Higher General Secondary Education (such as HAVO, Atheneum, Gymnasium)
- Higher Vocational Education (HBO)
- Scientific (university) education
- Other, namely _______

| 1. We would like to know if you have sought information during your illness and recovery period on the following (medical) topics.   *Please indicate what corresponds to your situation*  a. What is ... cancer?  b. What treatments are available  c. What the side effects may be during treatment  d. What complementary or alternative care options are available  e. Where to find a good doctor  f. What the best hospital is  g. What trials/research are in progress  h. What the treatment guidelines are  i. What the consequences (late effects) of treatment may be  j. About cancer and heredity  k. About fertility and wanting children after cancer  l. About metastases  m. About recurrence of the same cancer  n. About the chance of getting another cancer  o. How the patient can be actively involved in treatment choices  p. About the physician-patient relationship  q. Information on palliative treatments / palliative care  r. Figures on survival rates  s. Information about dying  t. Other, namely | Yes  O  O  O  O  O  O  O  O  O  O  O  O  O  O  O  O  O  O  O  O | No  O  O  O  O  O  O  O  O  O  O  O  O  O  O  O  O  O  O  O  O |
| --- | --- | --- |

| 1. We would like to know if you have ever sought information about the following (psychosocial) subjects during and after your treatment   *Please indicate what corresponds to your situation*  a. What someone can contribute to recovery  b. Information about intimacy and sexuality  c. Help in learning to cope with physical problems (e.g., fatigue, pain)  d. Improving satisfaction with one's own body image after treatment  e. About exercise and sports  f. About lifestyle and nutrition  g. About help with returning to study and/or work  h. About assistance in obtaining insurance and/or a mortgage  i. Help with financial problems  j. Meeting opportunities for AYA peers  k. About establishing relationships  l. About help for family members and friends  m. Effects on a young family  n. Help in learning to cope with mental health problems (e.g., anxiety and depression)  o. About coping with parents / family members  p. Learning to look at life in a positive way  q. Learning to stand up for yourself, regain self-confidence  r. How to deal with the feeling of being behind on "healthy" peers  s. About friendships  t. About spirituality  u. About religion  v. Other, namely | Yes  O  O  O  O  O  O  O  O  O  O  O  O  O  O  O  O  O  O  O  O  O  O | No  O  O  O  O  O  O  O  O  O  O  O  O  O  O  O  O  O  O  O  O  O  O |
| --- | --- | --- |

| 1. How often did you search for information about cancer on the Internet?   (Not all phases named below need to apply to you.)  BEFORE DIAGNOSE  a. When I had symptoms, but didn't know what it was yet  b. During the period when I was being examined in the hospital, but did not yet know that there was cancer  DIAGNOSE  c. After the specialist had told me that it was cancer  TREATMENT  d. During the period when I had to wait for the surgery/treatment  e. During the chemotherapy treatment  f. During the treatment with radiotherapy  g. During hormone therapy  AFTER TREATMENT (checks ups)  h. Since I am no longer being treated, but I still go to the doctor for check-ups  PALLIATIVE PHASE  i. Now that it is clear I cannot get better | Daily  O  O  O  O  O  O  O  O  O | Weekly  O  O  O  O  O  O  O  O  O | Monthly  O  O  O  O  O  O  O  O  O | Never  O  O  O  O  O  O  O  O  O | N/A  O  O  O  O  O  O  O  O  O |
| --- | --- | --- | --- | --- | --- |

| 1. What online options did you have and what wishes do you have? *Indicate on each line if something is possible and if it is (also) your wish* | Possible | | | | Need | |
| --- | --- | --- | --- | --- | --- | --- |
|  | Yes | Some­times | No | Don’t know | Yes | No |
| a. Being able to view one's own medical records  b. Being able to request results of examinations  c. Being able to email/e-consult with the treating physician(s)  d. Be able to email/e-consult with the nurses involved  e. Be able to request and/or renew prescriptions  f. Be able to request a diagnostic test  g. May ask for a referral to another specialist  h. Be able to make an appointment with one's own physicians  i. Be able to do self-diagnostic tests  j. Being able to "meet" online peers who are being treated at the same hospital  k. Being able to receive reminders to support treatment (e.g., for an appointment)  l. Being able to 'meet' face-to-face with peers who are being treated at the same hospital  m. Being able to suggest ideas for improving the treatment  n. Being able to keep track of things that bother you (such as fatigue)  o. Be able to receive personalized advice tailored to your symptoms  p. Be able to receive an overview of additional care options outside the hospital  q. Be able to rate a health care provider or health care facility  r. Be able to participate in an online self-help course  s. Room for any clarification: _____ | O  O  O  O  O  O  O  O  O  O  O  O  O  O  O  O  O  O | O  O  O  O  O  O  O  O  O  O  O  O  O  O  O  O  O  O | O  O  O  O  O  O  O  O  O  O  O  O  O  O  O  O  O  O | O  O  O  O  O  O  O  O  O  O  O  O  O  O  O  O  O  O | O  O  O  O  O  O  O  O  O  O  O  O  O  O  O  O  O  O | O  O  O  O  O  O  O  O  O  O  O  O  O  O  O  O  O  O |
