# Supplementary material for: AYAs' online information and eHealth needs: A comparison with healthcare professionals' perceptions
Source: Cancer Med. 2022 Jul 25;12(2):2016–26. doi: 10.1002/cam4.5048 (PMC9883566; doi:10.1002/cam4.5048)
Supplement: Supplementary file 2 — Appendix S2 [file CAM4-12-2016-s002.docx]

**Appendix B**

1. What is your age? _______
2. What is your gender?

- Woman
- Male

1. What is your occupation? (ordered alphabetically)

- Occupational physician
- Dietician
- Physiotherapist
- Social worker
- Medical specialist / specialty: _______
- Psychologist
- Sexologist
- Nursing specialist
- Nurse specialist / specialty: _______
- Other, namely: _______

‘

1. Are you working in an AYA knowledge center? (See: https://aya4net.nl/ayazorgnetwerk/)

- Yes
- No

1. Are you working in an AYA team?

- Yes
- No

1. Please tick from which topics you think are important for AYAs.

▢ What is ... cancer?

▢ What treatments are available

▢ What the side effects of treatment are

▢ What complementary care options are available

▢ Where to find a good doctor

▢ What the best hospital is

▢ What trials/research are in progress

▢ Treatment guidelines

▢ What the consequences (late effects) of treatment may be

▢ About cancer and heredity

▢ About fertility and desire for children after cancer

About metastases

About recurrence of the same cancer

About the chance of getting another cancer

▢ How AYA can be actively involved in treatment choices

▢ Physician-patient relationship

▢ Information about palliative care

▢ Figures on survival rates

▢ Information about dying

▢ Other, namely: _______

1. Of which topics do you think it is important to have age-specific information for AYAs during and after treatment?

▢ What a person can contribute themselves to recovery

▢ Information about intimacy and sexuality

▢ Help with learning to cope with physical problems (such as fatigue, pain)

▢ Improving body image after treatment

▢ About exercise and sports

▢ About lifestyle and nutrition

▢ About and help with returning to study and/or work

▢ About and assistance in obtaining insurance and/or mortgage

▢ Help with financial problems

▢ Meeting opportunities for AYA peers

▢ Information about forming relationships

▢ About help for family members and friends

▢ Effects on young family

▢ Help in learning to cope with mental health problems (e.g., anxiety and depression)

▢ About dealing with parents / relatives

▢ Learning to look at life in a positive way

Learning to stand up for themselves, regain self-confidence

▢ How to deal with the feeling of being behind "healthy" peers

▢ About friendships

▢ About spirituality

▢ About religion

▢ Other, namely: _______

1. Can you estimate how many of the AYAs search for information on the Internet?

|  | No one | Some | Many | All |
| --- | --- | --- | --- | --- |
| **BEFORE DIAGNOSIS**  In case of complaints  During investigation  **DIAGNOSE**  After being told it is cancer  **TREATMENT**  Period when AYA had to wait for surgery/treatment  During treatment with chemotherapy  During radiotherapy treatment  During hormone therapy treatment  **AFTER TREATMENT** (checks)  When AYA still sees the doctor for checks    **PALLIATIVE PHASE**  When it is clear that the AYA cannot get better | O  O  O  O  O  O  O  O | O  O  O  O  O  O  O  O | O  O  O  O  O  O  O  O | O  O  O  O  O  O  O  O |

1. What online options does the hospital offer to AYAs undergoing treatment at your hospital?

*Please tick on each line whether something is* ***possible*** *and whether you think AYAs perceive it as* ***needed****.*

|  | Possible | | | Need | |
| --- | --- | --- | --- | --- | --- |
|  | Yes | No | Don’t know | Yes | No |
| Access to personal medical information  Being able to request results of examinations  Being able to send emails (e-consult) with the doctor(s) treating you  Being able to send e-mails (e-consult) with the nurses involved  Be able to request and/or renew prescriptions  Be able to request diagnostic testing  Request a referral to another specialist  Be able to make an appointment with own doctors  Be able to do self-diagnostic tests  Being able to "meet" online peers who are being treated for cancer in the same organization  Being able to receive reminders to support treatment  Being able to 'meet' face-to-face with peers treated at the same hospital  Being able to suggest ideas for improving treatment  Being able to keep track of issues that trouble the AYA (such as fatigue)  Be able to receive personalised advice tailored to their symptoms  Be able to receive an overview of additional care options  Be able to rate a health care provider or health care facility  Be able to participate in an online self-help course  Be able to report complaints (legal) | O  O  O  O  O  O  O  O  O  O  O  O  O  O  O  O  O  O  O | O  O  O  O  O  O  O  O  O  O  O  O  O  O  O  O  O  O  O | O  O  O  O  O  O  O  O  O  O  O  O  O  O  O  O  O  O  O | O  O  O  O  O  O  O  O  O  O  O  O  O  O  O  O  O  O  O | O  O  O  O  O  O  O  O  O  O  O  O  O  O  O  O  O  O  O |
